# Metabolic oncology: clonal selection and the pre-existing origins of cancer resistance

**DOI:** 10.3389/fonc.2026.1951576

**Published:** 2026-10-16

**Authors:** Cesar A. Perez, Annette R. Khaled

**Affiliations:** 1 Sarah Cannon Research Institute at Florida Cancer Specialists & Research Institute, Lake Nona, University of Central Florida, Orlando, FL, United States; 2 Burnett School of Biomedical Sciences, College of Medicine, University of Central Florida, Orlando, FL, United States

**Keywords:** cancer metabolism, clonal selection, EHMT1/EHMT2, GLP-1 receptor agonist, treatment resistance

## Abstract

Treatment resistance remains the principal cause of cancer mortality, conventionally modeled as an adaptive response arising under therapeutic pressure. However, a substantial component of resistance is plausibly established before therapeutic exposure, determined by the metabolic environment of the tissue in which the malignancy arises. This article advances the hypothesis that chronic metabolic dysregulation constitutes a selective pressure acting on premalignant tissue. Hyperinsulinemia and increased bioavailable IGF-1 sustain PI3K–AKT–mTOR and RAS–MAPK signaling in tissues where insulin resistance has preserved mitogenic sensitivity, creating an environment permissive to clonal selection. Metabolic intermediates serve as obligate substrates for chromatin-modifying enzymes, coupling metabolic state to the epigenome across the years of subclinical dysregulation that precede diagnosis. Within this landscape, hypoxia stabilizes G9a and promotes locus-specific H3K9 methylation at apoptotic and differentiation loci, whereas one-carbon and S-adenosylmethionine status determine the available H3K9me1 substrate pool. The consequence is an elevated apoptotic threshold and enrichment of epigenetically primed, apoptosis-resistant cells before a tumor is histologically apparent or clinically detected. The argument is fundamentally Darwinian: the metabolic environment selects its survivors before any therapeutic intervention. Clonal dynamics subsequently attributed to therapy may therefore reflect, in part, selection of a pre-existing, resistance-primed population. This is advanced not as an alternative to clonal evolution, of which selection of pre-existing clones is a constituent process, but as a determinant of the pre-treatment resistance heterogeneity upon which therapeutic pressure acts. Existing data on metabolic intervention are discordant, and the discrepancies appear more consistent with differences in intervention potency, timing, and trial design than with failure of the proposed mechanism. Much of the supporting evidence remains indirect and derives from heterogeneous experimental settings. The observations that would distinguish this framework from reverse causation and treatment-induced plasticity are specified, together with its central untested prediction: that pretreatment metabolic state correlates with baseline subclonal architecture, chromatin state, and treatment outcome.

## Introduction

Cancer does not simply arise—it is selected. Long before a pathologist identifies a malignant cell, the tissue microenvironment has been conducting a silent Darwinian competition: metabolically stressed cells acquire epigenetic modifications that raise apoptotic thresholds and promote constitutive survival signaling, and those that do are enriched relative to their neighbors. By the time the oncologist sees the patient, that process may be largely complete. What we call treatment resistance may often be less a response to therapy than the clinical unveiling of a cellular architecture established years earlier.

Despite extensive paradigm shifts over the last two decades, three problems continue to define the limits of modern oncology: prevention that operates on single pathways while cancer develops through a convergent process shaped by multiple forces across decades; recurrence after curative-intent therapy at rates that genomic profiling has not learned to predict; and treatment resistance, the dominant cause of cancer mortality, which we can characterize in molecular detail but cannot yet prevent. These three problems may share a common upstream contributor—the chronic metabolic environment in which tumors originate, evolve, and, in too many patients, persist despite treatment.

## The metabolic environment as selective pressure

The metabolic environment in which a tumor develops is not background noise. Chronic hyperinsulinemia increases bioavailable IGF-1 (insulin-like growth factor 1) and sustains activation of the PI3K–AKT–mTOR (phosphoinositide 3-kinase–protein kinase B–mammalian target of rapamycin) and RAS–MAPK (mitogen-activated protein kinase) cascades in tissues where insulin resistance has paradoxically preserved mitogenic sensitivity, thereby creating a tissue environment highly permissive to clonal selection (1). Elevated inflammatory mediators, including interleukin-6, leptin, and adipokines, further amplify proliferative signaling while attenuating apoptotic responses. A systematic review using the hyperinsulinemic–euglycemic clamp confirmed that severe insulin resistance is present in patients with cancer relative to matched controls (mean glucose disposal rate 4.7 vs. 7.5 mg/kg/min; *p* < 0.01), with limited evidence suggesting that this adaptation may precede clinical diagnosis (2). This is not a risk factor in the conventional epidemiologic sense, but an ongoing selective pressure operating across the years of premalignant evolution that precedes any clinically detectable disease.

This environment shapes the cellular phenotype substantially through the epigenome: metabolic intermediates—S-adenosylmethionine (SAM), acetyl-CoA, α-ketoglutarate, and NAD^+^—serve as obligate substrates for chromatin-modifying enzymes, continuously coupling the cell’s metabolic state to its epigenetic landscape (3–5). Changes in one-carbon metabolism can directly alter histone methylation and downstream transcription across every cell division that occurs during years of subclinical metabolic dysregulation.

Among the relevant modifications, histone H3 lysine 9 (H3K9) methylation is catalyzed by the methyltransferases EHMT1 and EHMT2 (GLP and G9a; distinct from glucagon-like peptide-1), which deposit the mono- and dimethyl marks (H3K9me1 and H3K9me2) using SAM as the methyl donor (6). H3K9me2 at apoptotic and differentiation loci has been linked to drug resistance (7, 8).

Hypoxia is the driver of H3K9me2 gain: it stabilizes G9a protein and increases H3K9me2 at tumor-suppressor and apoptotic loci (9). SAM availability works separately: when SAM is limiting, EHMT1/2 preferentially preserve H3K9me1 while higher-order di- and trimethylation decline, so SAM depletion does not itself add to H3K9me2 (5). One-carbon/SAM status is therefore best understood as setting the H3K9me1 substrate pool, whereas hypoxia-stabilized G9a generates the resistance-associated H3K9me2. Whether G9a regulation in premalignant epithelial compartments mirrors the dynamics seen in metabolic tissues like liver and adipose, where G9a expression drops under high-fat conditions, has not been systematically characterized. It is a distinction the framework requires but the data have not yet resolved.

A related precedent comes from pancreatic cancer, where pharmacologic inhibition of the metabolic pathway driving epigenomic reprogramming reversed both the chromatin state and the malignant phenotype. That is proof of concept that metabolic intervention can undo what epigenetic hardening has installed (10).

The clinical prediction converges with the epigenetic argument: tumors arising in metabolically dysregulated tissue should harbor larger pre-existing resistant subclone fractions—parallel mechanisms, not competing ones, producing a cell population more likely to progress, survive dormancy, and resist treatment (**Figure 1**).

**Figure 1:**
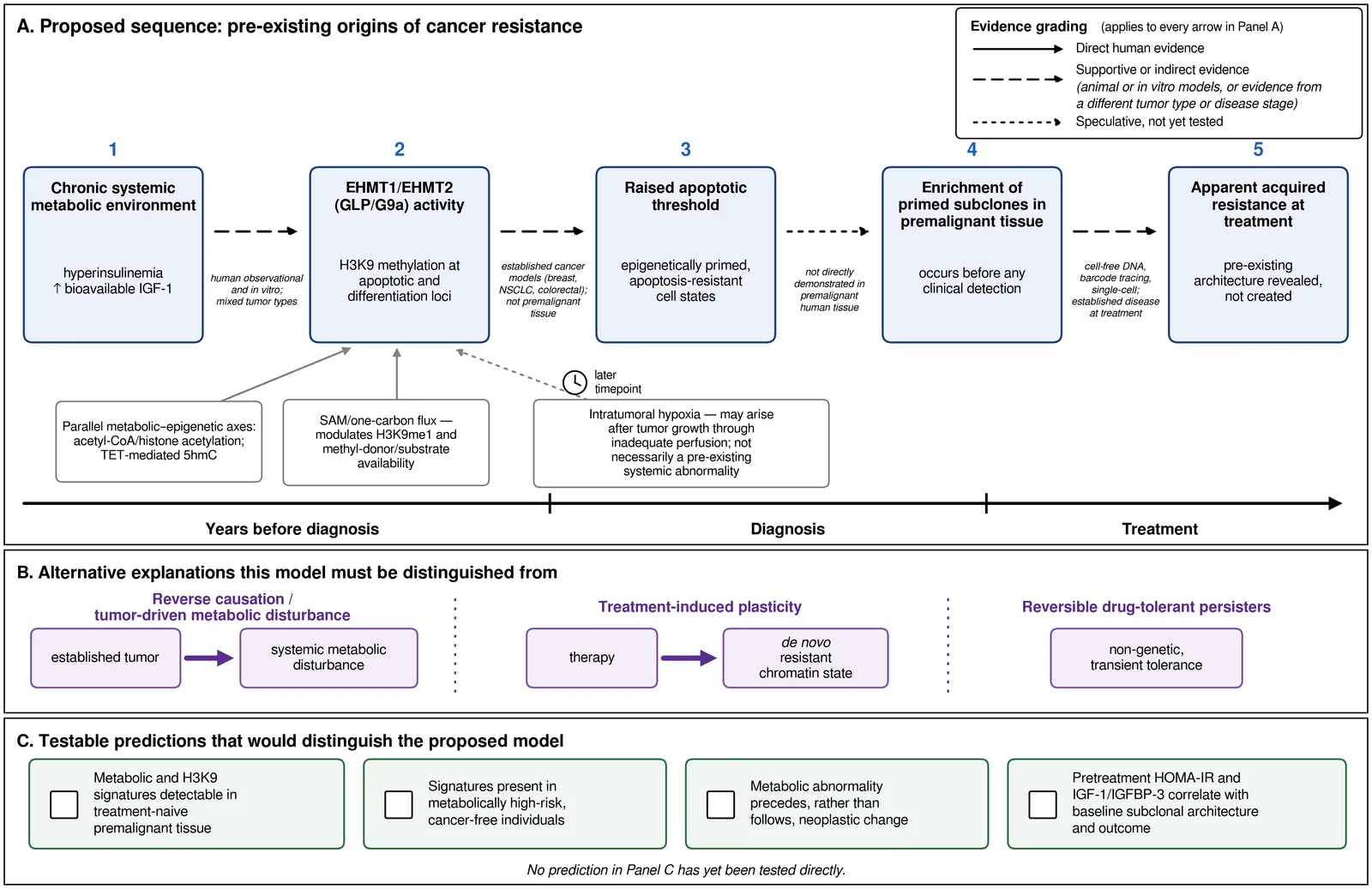
A hypothesis-generating model in which chronic metabolic dysregulation promotes EHMT1/EHMT2-dependent apoptotic priming before clinical detection. **(A)** Proposed causal chain. Chronic hyperinsulinemia and increased bioavailable IGF-1 (box 1) engage insulin/IGF-1 receptor signaling in premalignant tissue. Hypoxia-stabilized G9a, together with the H3K9 methyltransferase activity of EHMT1 and EHMT2 (GLP and G9a, respectively; GLP here denotes the EHMT1 gene product, distinct from glucagon-like peptide-1), promotes H3K9 methylation at pro-apoptotic and differentiation loci (box 2), raising the apoptotic threshold (box 3) and enriching epigenetically primed, apoptosis-resistant cells before disease is clinically detectable (box 4). Resistance observed at treatment (box 5) is therefore revealed by therapy, through selection of a pre-existing primed state, rather than created by it. Arrows are graded by evidentiary status: solid, direct human evidence; dashed, supportive or indirect evidence from animal or *in vitro* models or from a different tumor type or disease stage; dotted, speculative and not yet tested. Italic annotations state the biological setting of the supporting evidence. Grey elements are secondary inputs, indicating that EHMT1/EHMT2 is one convergent node rather than the sole determinant; one-carbon and S-adenosylmethionine status modulates H3K9me1 and methyl-donor availability, and intratumoral hypoxia is drawn as a temporally later, parallel input because it may arise after tumor growth through inadequate perfusion. Most support for the box 2–box 5 links derives from established breast, non-small-cell lung, and colorectal cancer models rather than premalignant tissue; enrichment of primed cells in premalignant tissue (box 4) is the model’s principal untested step. **(B)** Alternative explanations from which the model must be distinguished: reverse causation or tumor-driven metabolic disturbance, treatment-induced plasticity, and reversible drug-tolerant persister states. **(C)** Predictions that would discriminate the model from these alternatives. None has yet been tested directly.

### The GLP-1RA signal and what it reveals

The implications for cancer prevention are where the population-scale data have arrived first. GLP-1 receptor agonists (GLP-1RA; glucagon-like peptide-1 receptor agonists, not to be confused with GLP, the G9a-like protein EHMT1 discussed above), which reduce hyperinsulinemia, attenuate IGF-1 bioavailability, and suppress systemic inflammatory markers (11, 12), are currently the most informative pharmacologic probe of the metabolic hypothesis, not its therapeutic endpoint. The emerging observational signal is provocative: a 2026 meta-analysis of 24 observational studies enrolling nearly 4 million patients found a 30% reduction in obesity-related cancer risk (13); in addition, a large retrospective cohort confirmed reduced overall cancer incidence in adults with obesity [hazard ratio (HR) 0.83; 95% confidence interval (CI), 0.76 to 0.91] (14). Body mass index (BMI)-independent reductions in acute myeloid leukemia and breast cancer incidence, persisting after adjustment for metabolic covariates, suggest a mechanism operating through metabolic signaling rather than adiposity reduction alone, though these data remain preliminary conference abstracts (15, 16). In a preclinical colitis-associated carcinogenesis model, tirzepatide reduced tumor burden by suppressing metabolic pathways without inducing weight loss (17).

The most compelling recent clinical evidence comes from a retrospective cohort of 841,831 patients with breast cancer drawn from the TriNetX network, in which propensity-matched GLP-1RA users demonstrated approximately 60% lower all-cause mortality at both 5 and 10 years compared with non-users, with a large relative improvement in recurrence-free survival (18). A finding of this magnitude in a dataset of this size commands attention and scrutiny, all the more so given the retrospective nature of the analysis. Interestingly, when GLP-1RA users were compared with sodium-glucose cotransporter 2 (SGLT2) inhibitor users, another drug class that corrects insulin resistance and improves metabolic signaling through a distinct mechanism, and not untreated controls, the survival difference was attenuated, with only modest residual benefit after covariate adjustment (18). Thus, if two mechanistically distinct agents that share the common property of metabolic normalization produce convergent oncologic signals, then the operative variable might not be the drug, but the metabolic environment that the drugs correct.

This inference does not come without a counterweight. A meta-analysis of 48 randomized controlled trials (RCTs) enrolling 94,245 participants found no effect of GLP-1RAs on obesity-related cancer incidence (19, 20). A separate RCT-based meta-analysis reported increased thyroid and colorectal cancer risk with GLP-1RA use (Mantel–Haenszel odds ratio (MH-OR) 1.55 and 1.27, respectively) (21). Trials were not designed with cancer endpoints and had inadequate follow-up durations for solid tumor latency, and metabolically enriched patient selection was absent. However, these are observations about trial design, not exculpations of the null result. The metformin precedent is instructive here: the MA.32 trial and a subsequent meta-analysis of 22 randomized trials confirmed no benefit on disease-free or overall survival (22, 23). The mechanistic rationale for metformin was compelling; the intervention was simply not potent enough. GLP-1RA investigators must reckon with that history—not as a reason for pessimism, but as a specification: to meaningfully reverse the metabolic selective pressure and achieve clinically tangible outcomes, a more potent, broader-acting intervention deployed in a metabolically high-risk population will be required.

## Metabolic selection of pre-existing resistance

The somatic evolution literature indicates that resistance predominantly pre-exists. This view is supported by multiple orthogonal approaches, including cell-free DNA analysis, high-complexity barcode tracing, and single-cell sequencing, which have collectively demonstrated that acquired resistance frequently arises from pre-existing subclones selected by therapeutic pressure. Selection of pre-existing clones is itself a component of clonal evolution rather than an alternative to it. Furthermore, the same single-cell analyses that identify pre-existing resistant genotypes also document treatment-associated transcriptional reprogramming, indicating that selection and adaptation operate together rather than exclusively (24–26).

What has been missing is a specific account of how that resistant architecture becomes established in the first place. The metabolic framework proposed here provides that account. Chronic metabolic stress (driven by tissue hypoxia, altered one-carbon flux, and excess growth-factor signaling) creates the conditions in which EHMT1/2 have the upper hand in premalignant tissue. As other chromatin marks fade, this one holds on, concentrating at genes that govern cell death and differentiation. The result is a subpopulation of cells already primed to resist apoptosis and keep growing, selected before a tumor is histologically apparent, clinically detected, or any treatment is given. These clones might not be generated by treatment, but revealed by it. The mechanism proposed here acts principally on epigenetically primed but genetically uncommitted cell states and may contribute to the pool of pre-existing genetic clones; it is not intended to account for reversible drug-tolerant persister states or to exclude resistance genuinely acquired during treatment.

The experiment that would directly test this hypothesis has not been performed. Pre-treatment fasting insulin, homeostatic model assessment of insulin resistance (HOMA-IR), and IGF-1/insulin-like growth factor binding protein 3 ratio should be correlated with baseline subclonal architecture, H3K9me2 state, circulating tumor DNA (ctDNA) heterogeneity, and treatment outcome in metabolically unselected patients initiating systemic therapy. This is a tractable, fundable, immediately actionable study design, and the central ask of this framework.

We acknowledge that most of the supporting evidence is indirect and drawn from heterogeneous models. Reverse causation, in which an established tumor drives systemic metabolic disturbance, predicts that metabolic abnormality follows neoplastic change; our model requires the opposite ordering. Treatment-induced plasticity predicts that resistant chromatin states emerge only after therapeutic exposure; our model predicts that they are demonstrable in treatment-naive tissue. Each of these predictions is testable, and none has yet been tested directly. Where evidence is currently indirect, we have labeled it as supportive or speculative rather than direct.

Perhaps no population makes the argument more urgent than young adults (≤45 years) with early-onset colorectal cancer who are neither obese nor diabetic, a cohort for whom neither genetic nor conventional metabolic risk factors suffice as explanation. Subclinical hyperinsulinemia, IGF-1 axis activation, and metabolic stress-driven epigenetic changes (potentially including EHMT1/2-mediated chromatin remodeling) may constitute the operative carcinogenic sequence even when standard screens appear normal (27, 28). That the metabolic environment is not merely a risk modifier but an active driver of oncologic outcomes in colorectal cancer is no longer entirely inferential: the CHALLENGE trial, a phase III randomized study, demonstrated a significant disease-free survival advantage with structured post-chemotherapy exercise. This intervention, known to reduce fasting insulin, IGF-1 bioavailability, and systemic inflammatory markers, provides the first level I evidence, in stage III and high-risk stage II colon cancer, that an intervention operating through these same metabolic axes improves oncologic outcomes in this disease (29). The mechanism of that benefit remains incompletely understood, and that is precisely the point.

A specific caution is warranted in the neoadjuvant setting. In a retrospective multicenter cohort of 343 patients with early-stage triple-negative breast cancer receiving KEYNOTE-522, concurrent GLP-1RA use was associated with reduced pathologic complete response rates (pCR 30.8% vs. 65% in controls; *p* < 0.001); only 26 patients were GLP-1RA users, and GLP-1 exposure did not retain significance in multivariate analysis (30). A subsequent peer-reviewed multi-institution study of 374 patients receiving the same regimen found that diabetes mellitus, not GLP-1RA use, was independently associated with reduced pCR (*p* = 0.03), alongside high tumor grade (31). Whether the metabolic state, its pharmacologic treatment, or their interaction drives the observed pCR reduction remains unresolved. What is clear is that concurrent GLP-1RA use during cytotoxic chemotherapy requires prospective evaluation. Trials should stratify by GLP-1RA exposure timing, diabetes status, and metabolic phenotype, and incorporate pre-specified exploratory resistance endpoints alongside established primary endpoints such as pCR and event-free survival.

## Discussion

For many malignancies, an underweighted component of the problem may be metabolic selection: the pre-treatment enrichment of aggressive, epigenetically primed cells by the metabolic environment in which the tumor arose. We do not offer this as a replacement for clonal evolution, of which the selection of pre-existing clones is a constituent process, but as a proposed contributor to the pre-treatment resistance heterogeneity on which subsequent therapeutic pressure acts. The emphasis is not merely semantic. It reframes the therapeutic objective from attacking a disease that will inevitably generate resistance to modifying the environment that selected for resistance in the first place, potentially maintaining otherwise aggressive clones in a quiescent, treatment-sensitive state. The convergence of two mechanistically distinct agents, GLP-1 receptor agonists and SGLT2 inhibitors, on overlapping oncologic signals points away from receptor-specific biology and toward metabolic correction as the operative variable, strengthening the argument that what is being modified is the selective pressure, not a single pathway within it. This is, at its core, a Darwinian argument: the metabolic environment may select for resistance long before any therapeutic intervention.

The thesis is not that GLP-1 receptor agonists prevent or treat cancer; the evidence does not yet support that claim. It is that metabolic dysregulation constitutes a modifiable, systematically under-addressed selective pressure linking prevention, recurrence, and resistance through a coherent mechanism. Patients with established metabolic indications should be treated for those indications on their own merits, independent of any presumed oncologic benefit. Neither this manuscript nor the available evidence establishes that GLP-1 receptor agonists prevent recurrence or resistance, but prospective trials enrolling metabolically high-risk patients with cancer endpoints and adequate follow-up are needed to resolve the tension between the observational signal and the randomized null. The answers will matter, whatever they turn out to be.
